# Unmet needs for healthcare and social support services in patients with Huntington’s disease: a cross-sectional population-based study

**DOI:** 10.1186/s13023-015-0324-8

**Published:** 2015-09-28

**Authors:** Marleen R. van Walsem, Emilie I. Howe, Kristin Iversen, Jan C. Frich, Nada Andelic

**Affiliations:** Centre for Habilitation and Rehabilitation Models and Services (CHARM), Institute for Health and Society, University of Oslo, P.O. Box 1130, Blindern, 0318 Oslo Norway; Department of Neurohabilitation, Oslo University Hospital, P.O. Box 4950, Nydalen, 0424 Oslo Norway; Department of Physical Medicine and Rehabilitation, Oslo University Hospital, P.O. Box 4950, Nydalen, 0424 Oslo Norway; Centre for Rare Disorders, Oslo University Hospital, Rikshospitalet, P.O. Box 4950, Nydalen, 0424 Oslo Norway; Department of Health Management and Health Economics, University of Oslo, P.O. Box 1130, Blindern, 0318 Oslo Norway; Department of Neurology, Oslo University Hospital, P.O. Box 4950, Nydalen, 0424 Oslo Norway

**Keywords:** Huntington’s disease, Healthcare services, Social support services, Healthcare needs

## Abstract

**Background:**

In order to plan and improve provision of comprehensive care in Huntington’s disease (HD), it is critical to understand the gaps in healthcare and social support services provided to HD patients. Research has described utilization of healthcare services in HD in Europe, however, studies systematically examining needs for healthcare services and social support are lacking. This study aims to identify the level and type of met and unmet needs for health and social care services among patients with HD, and explore associated clinical and socio-demographic factors.

**Methods:**

Eighty-six patients with a clinical diagnosis of HD living in the South-Eastern region of Norway were recruited. Socio-demographic and clinical characteristics were collected. The Needs and Provision Complexity Scale (NPCS) was used to assess the patients’ needs for healthcare and social services. Functional ability and disease stage was assessed using the UHDRS Functional assessment scales. In order to investigate factors determining the level of total unmet needs and the level of unmet needs for Health and personal care and Social care and support services, multivariate logistic regression models were used.

**Results:**

A high level of unmet needs for health and personal care and social support services were found across all five disease stages, but most marked in disease stage III. The middle phase (disease stage III) and advanced phase (disease stages IV and V) of HD increased odds of having a high level of total unmet needs by 3.5 times and 1.4 times respectively, compared with the early phase (disease stages I and II). Similar results were found for level of unmet needs in the domain Health and personal care. Higher education tended to decrease odds of high level of unmet needs in this domain (OR = 0.48) and increase odds of higher level of unmet needs in the domain of Social care and support (OR = 1.3). Patients reporting needs on their own tended to decrease odds of having unmet needs in Health and personal care (OR = 0.57).

**Conclusions:**

Needs for healthcare and social services in patients with HD should be assessed in a systematic manner, in order to provide adequate comprehensive care during the course of disease.

**Electronic supplementary material:**

The online version of this article (doi:10.1186/s13023-015-0324-8) contains supplementary material, which is available to authorized users.

## Background

Many rare diseases, such as Huntington’s disease (HD), are chronic and complex, and are associated with physical, mental or neurological disabilities. Systematic assessment of patients’ needs for healthcare and social services may identify gaps that could lead to improved service provision [1, 2].

HD is an autosomal dominant neurological disease caused by an expanded CAG repeat in the Huntingtin gene. The disease is characterized by progressive functional decline and motor, psychiatric and cognitive symptoms, in addition to weight loss, sleep disturbances and dysregulation of the autonomic nervous system [3–6]. A clinical diagnosis of HD is given when unequivocal motor symptoms are present, but subtle motor signs, psychiatric symptoms and cognitive changes may be present years prior to clinical diagnosis [7–11]. Clinical symptoms of HD usually develop during adult life between 30 and 50 years of age and disease duration from first clinical symptoms to complete care dependency and death is approximately 15–20 years [4].

At present, there is no curative treatment for HD, and treatment is aimed at alleviating symptoms, maintaining and improving function and quality of life [12]. HD patients in early to middle stages of the disease need coordinated multidisciplinary healthcare services, including assessment of cognitive function and counselling by (neuro)psychologists [4], rehabilitation programs [13, 14], active physiotherapeutic interventions [15, 16], speech therapist training [4, 17, 18] and occupational therapy [4]. Patients in advanced stages of the disease are usually dependent on full-time personal care, but may still benefit from multidisciplinary care [19]. Family members experience challenges and need support and guidance [20].

Although there is general agreement that comprehensive, multidisciplinary care is needed [4, 19, 21–24], the complex and changing clinical picture may be a challenge for health professionals. Standards of care, aimed at separate groups of healthcare professionals (i.e., speech and language therapists) have been published, with the purpose of being a foundation for further research and evaluation of provided care [18, 25, 26]. A few clinics adapting comprehensive care models have emerged in the US, Australia and Europe [4, 24, 27].

In order to plan and improve provision of long-term care in HD, it is essential to understand what healthcare and social services HD patients receive, and what unmet healthcare needs they may have. A few studies have assessed health and social care utilization and needs in HD and results showed a number of unmet needs related to body functions, activities and level of participation as well as carer support [28, 29].

To date, research has not addressed healthcare needs of HD patients in Norway. Norway, like the other Scandinavian countries, is a welfare state with equal access to health and social care services, and services are either free or subsidized at point of delivery. Thus, the Scandinavian studies on HD patients and delivery of healthcare services may be of international interest.

The aims of the present study are threefold: a) to investigate the level of unmet needs for healthcare and social support services among HD patients, b) to investigate how the level of unmet needs are divided across disease phases, and c) to investigate the association between socio-demographic and clinical disease characteristics and levels of unmet needs.

We anticipated considerable levels of unmet needs for healthcare and social support services across all phases of the disease. Furthermore, we anticipated the advanced disease phase to be highly associated with the level of unmet needs for healthcare and social support services.

## Methods

### Participants and participant recruitment

Patients with a clinical diagnosis of HD residing in the South-Eastern region of Norway, a region with a population of 2.7 million, were invited to participate in a survey of healthcare needs and utilisation of healthcare services. Eligible patients were identified through the Department of Neurology, Department of Neurohabilitation, and Department of Medical Genetics at Oslo University Hospital, the regional academic medical center. In addition, patients were recruited through the Centre for Rare Disorders at Oslo University Hospital, a national advisory service for HD that offers guidance to patients, families and healthcare professionals. In a further effort to reach all eligible patients, we collaborated with a Norwegian professional network for community care in HD (Huntington fagnettverk) and the Norwegian HD lay association (Landsforeningen for Huntingtons sykdom). The Vikersund Rehabilitation Centre, which runs a rehabilitation program for HD patients, were informed about the study and issued invitations to additional patients.

All eligible patients received a written invitation, enclosing information about the study and an informed consent form. Following return of the consent form, the patient/carer was contacted and an appointment for a study visit was made.

**Fig. 1:**
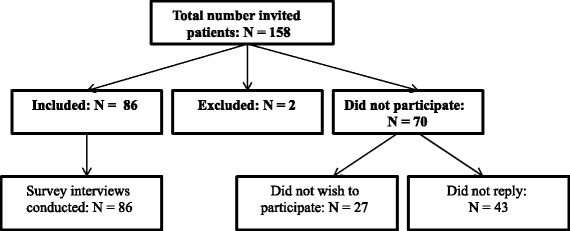
Flow chart illustrating patient recruitment process

We identified a total of 158 eligible patients (which correspond to a prevalence of 5.9/100.000) who were invited to participate in the study, of which 88 patients gave their consent to participate and were included. Among the 70 patients who were not included, 27 declined to participate and 43 did not reply (see Fig. 1 for flow chart illustrating patient recruitment). An expert HD clinician reviewed medical records if there was any doubt about the diagnosis. Two patients did not have sufficient symptoms to formally have been given a clinical diagnosis of HD and were therefore excluded. Finally, 86 (54.4 %) out of the 158 potential participants were included in the data-analysis.

### Ethics

The study was approved by the Regional Ethical Committee (ref. 2013/2089). Informed consent was obtained for all patients. For patients who were unable to give informed consent themselves, consent was obtained from the primary caregiver or legal representative.

### Data collection

Data were collected through interviews conducted by two experienced clinical raters, either during an outpatient study visit (38.0 %) or at the patients’ home (62.0 %). We recorded with whom the interview was performed, i.e., patient alone, patient with primary informal and/or formal carer, or informant only. Socio-demographic information and clinical functional assessment was recorded at the beginning of the visit. Co-morbid conditions not related to HD were also recorded. Furthermore, patients were rated regarding functional ability and needs for healthcare and social services. Patients’ medical records were reviewed if further information was needed.

### Description of measurements

#### Unified Huntington’s disease rating scale – functional assessment

The UHDRS-Functional assessment comprises three scales: a) the Total Functional Capacity Scale (UHDRS-TFC), rating ability to engage in occupation, manage finances and domestic chores, and perform activities of daily living (ADL), with a score range of 0 – 13. The scale is used to classify patients into five functional disease stages, using the following convention: A TFC score of 11–13 represents Stage I, a TFC score of 7–10 represents Stage II, TFC score of 3–6 represents Stage III, a TFC score of 1–2 represents Stage IV and a TFC score of 0 represents Stage V. b) the Functional Assessment Scale (FAS), a daily living checklist with scoring range 0–25, and c) the Independence scale (IS) with range 10 to 100. Higher scores indicate better functioning [30].

#### The Needs and Provision Complexity Scale (NPCS)

The NPCS was specifically developed to identify healthcare and social support needs among patients with long term neurological conditions in the UK [2]. It is a brief and practical tool for measuring the *needs* for healthcare and social support of an individual, and the extent to which those needs are *met* through service provision. At the individual level, the NPCS can be used to monitor the changing needs of patients over time and services provided to support them along the care pathway, while at a population level it can identify gaps of service provision. The NPCS clinician version consists of a 15-item measure with six sub-scales and a total scoring range of 0–50 covering “low” to “high” levels of needs. It has two parts: a) Part A *(NPCS-Needs)* which is completed by the clinician to evaluate each patients’ need for health and social care, b) Part B *(NPCS-Gets)* is a mirror image of the same instrument to evaluate the services that have been provided. Furthermore, the NPCS consists of two main domains a) Health and Personal care needs (score range 0–25), including the following subscales: Healthcare (score 0–6), Personal care score (0–10), Rehabilitation (0–9), and b) Social care and support needs (score range 0–25), including subscales Social and family support (score 0–13), Equipment (score 0–3) and Environment (score 0–9). For an overwiew of the NPCS, see Additional file 1.

The NPCS was translated by native speakers from English to Norwegian and then back-translated to English to check for accuracy. The translation was reviewed by expert researchers and clinicians in the field of healthcare services and HD.

### Statistical analyses

#### Outliers and missing values

Prior to analysis, the data were screened for extreme values and missing data. Some outliers were identified in the data on sample characteristics and NPCS scores, and checked with the original data material. These were true values and therefore kept in the statistical analyses. Initially, four missing values for variables years of education, level of education (lower vs. higher), and three for occupation type (manual vs. non-manual) were identified and later estimated from the patients’ medical records. For four patients, we were unable to collect information about disease duration (number of years with clinical diagnosis of HD) during the survey interview, and we used clinical information that was available through medical records to estimate disease duration. For three patients we were unable to attain CAG repeat number. Additionally, information on one item in the NPCS scale was missing (<2 %) for one participant.

#### Descriptive analyses

Descriptive statistics were used (proportions, mean values, standard deviations (SD) or median values with interquartile range, (IQR)) in order to describe the socio-demographic and clinical disease characteristics of the complete sample and across the five disease stages. Cross tabulations’ Chi-square tests were used in order to compare nominal socio-demographic and clinical characteristics across disease stages. Group comparisons across disease stages for continuous variables, were calculated using Non-parametric Kruskal-Wallis k-sample tests, as the data were not normally distributed (with exception of participants age).

#### Descriptive analyses for NPCS needs, gets and unmet needs

Scores for levels of *unmet needs* (representing gaps between patients’ needs for healthcare and social support services and provision of these services) were calculated as the discrepancy between the scores for level of the patients’ *Needs* and *Gets* (Score on NPCS Needs – Score on NPCS Gets = NPCS Unmet needs). The level of *unmet needs* were calculated for the *NPCS Total score*, for the domain scores *Health and personal care* and *Social care and support*, as well as for the six corresponding subscales. Descriptive statistics presented by median values with interquartile scores were used in order to present the level of patients’ needs, the level of provision (Gets), and the level of *unmet needs* for the total sample as well as for disease stage I-V. Additionally, frequencies and proportions of patients with *unmet needs* on each of the NPCS scores were calculated for the total sample and five disease stages. Group comparisons across the five disease stages for levels of needs, gets and unmet needs and for frequencies were made using Kruskal-Wallis k sample test. The *p*-value was set at 0.05.

#### Evaluating effects on the level of unmet needs

In order to investigate the factors determining the level of total unmet needs, and the level of unmet needs for Health and personal care and Social care and support services, multivariate logistic regression models were used. Regression analyses were performed on the group of patients having unmet needs on the *NPCS total score* and the two main domains *health and personal care* and *social care and support*. The NPCS total score and the domain scores were categorized into two groups based on median value: low level of unmet needs vs. high level of unmet needs. This resulted in the following categories: *NPCS Total unmet needs*: low level (1–6) vs. high level of unmet needs: >6); *NPCS Health and personal care unmet needs*: Low level (1–3) vs. high level (>3); *NPCS social care and support unmet needs*: Low level (1 & 2) vs. high level (>2). Due to the small number of patients, the five disease stages were collapsed into three disease *phases*: early phase (disease Stage I & II), middle phase (Stage III) and advanced phase (Stages IV & V) in the regression analyses. Statistically significant factors (level of education (lower vs. higher), informant (patient alone vs. patient and informant/informant alone) and disease phase (early, middle or advanced) from the univariate analyses including simple logistic regression were included in the multivariate models in order to investigate their impact on the total level of unmet needs and unmet needs for health and personal care and social care and support. We applied similar multivariate models to demonstrate that certain factors are common and consistently important. To control for the heterogeneity in the included sample, all models were adjusted for age (years), disease duration (years), and comorbidity (yes/no). Results from the multiple logistic regression analyses are presented with odds ratios (OR) with 95 % confidence intervals. Furthermore, the Hosmer-Lemeshow goodness-of-fit statistics were computed. Prior to conducting the logistic regressions we investigated multicollinearity. The variables with correlation coefficients > .70 were not entered in the regression analyses. All analyses were conducted using SPSS version 21.0; SPSS Inc. Chicago IL.

## Results

### Socio-demographic and clinical description of the patients

The socio-demographic characteristics for the total sample and across disease stages are summarized in Table 1. The median age was 57.5 years, and 54.7 % of the patients were men. The majority of patients lived at home (62.8 %). Of the complete sample of 86 patients, 12 (14 %) were in stage I, 23 (26 %) in stage II, 19 (22 %) in stage III, 15 (18 %) in stage IV, and 17 (20 %) in stage V. Overall, significant group differences across disease stages were found for variables Occupational situation, Housing situation and Informant.

**Table 1:** Socio-demographic statistics for complete sample and divided across V disease stages

		Complete sample (*N* = 86)	Stage I (*n* = 12)	Stage II (*n* = 23)	Stage III (*n* = 19)	Stage IV (*n* = 15)	Stage V (*n* = 17)	
Variables	Categories	Median (IQR)	Median (IQR)	Median (IQR)	Median (IQR)	Median (IQR)	Median (IQR)	Sign
Age^a^		58 (15)	49 (20)	54 (21)	58 (11)	58 (8)	59 (16)	0.104
Education (years/)^b^		12 (5)	13 (5)	12 (7)	11 (2)	12 (6)	11 (6)	0.168
		n (%)	n (%)	n (%)	n (%)	n (%)	n (%)	*P*-value (2sided)
Gender	Female	39 (45)	5 (42)	9 (39)	7 (37)	8 (53)	10 (59)	0.625
	Male	47 (55)	7 (58)	14 (61)	12 (63)	7 (47)	7 (41)	
Education	Lower	52 (60.5)	5 (42)	12 (52)	15 (79)	9 (60)	11 (65)	0.424
	Higher	34 (39.5)	7 (58)	11 (48)	4 (21)	6 (40)	6 (35)	
Sivil status	Single	36 (42)	4 (33)	7 (30)	9 (47)	8 (53)	8 (47)	0.587
	Married	50 (58)	8 (67)	16 (70)	10 (53)	7 (47)	9 (53)	
Occupation^c^	Manual	41 (48)	5 (42)	10 (43.5)	12 (63)	6 (40)	8 (47)	0.666
	Non-manual	42 (49)	7 (58)	13 (56.5)	7 (37)	8 (53)	7 (41)	
Occupational status	Employed	14 (16)	11 (92)	3 (13)	0 (0)	0 (0)	0 (0)	0.001
	Unemployed	72 (84)	1 (8)	20 (87)	19 (100)	15 (100)	17 (100)	
Informant	Patient	27 (31)	9 (75)	14 (61)	4 (21)	0 (0)	0 (0)	0.001
	Patient & informant/informant only	59 (69)	3 (25)	9 (39)	15 (79)	15 (100)	17 (100)	
Housing situation	Living at home	54 (63)	12 (100)	23 (100)	13 (68)	6 (40)	0 (0)	0.001
	Not living at home	32 (37)	0 (0)	0 (0)	6 (32)	9 (60)	17 (100)	
Residence	Rural	13 (15)	1 (8)	4 (17)	2 (10.5)	3 (20)	3 (18)	0.878
	Urban	73 (85)	11 (92)	19 (83)	17 (89.5)	12 (80)	14 (82)	

*IQR* Interquartile range; Group comparison across the five disease stages performed using Chi-square tests for independent samples (categorical values). ^a^normally distributed and therefore reported result from ANOVA). ^b^not normally distributed therefore performed and reported Kruskal-Wallis test. ^c^3 responses missing (1 in stage IV and 2 in stage V). Remaining proportions and comparisons are crosstabs / Chi-square. IQR: Interquartile range

Clinical characteristics for the total sample of patients across disease stages are presented in Table 2. The median values for disease duration, total FAS score and FAS independence scores were respectively 6.1 (IQR 6.8) years, 15 (IQR 17), 70 (IQR 35). Of the complete sample, 36 (42 %) patients had comorbid conditions, 5 (42 %) in disease stage I, 13 (56 %) in disease stage II, 10 (53 %) in disease stage III, 4 (27 %) in disease stage IV, and 4 (23 %) in disease stage V.

**Table 2:** Sample clinical characteristics

		Complete sample (*N* =86)	Stage I (*n* = 12)	Stage II (*n* = 23)	Stage III (*n* = 19)	Stage IV (*n* = 15)	Stage V (*n* = 17)	
Variables		Median (IQR)	Median (IQR)	Median (IQR)	Median (IQR)	Median (IQR)	Median (IQR)	Sign
Disease duration		6 (7)	2 (2)	5 (6)	7 (5)	8 (7)	10 (8)	*P* < 0.001
Total FAS score		15 (17)	24 (2)	20 (2)	15 (4)	5 (2)	0 (3)	*P* < 0.001
Independence score		70 (35)	97 (9)	80 (5)	65 (10)	45 (20)	20 (6)	*P* < 0.001
		n (%)	n (%)	n (%)	n (%)	n (%)	n (%)	*P*-value (2-sided)
Comorbid conditions								0.157
	None	50 (58)	7 (8)	10 (12)	9 (10)	11 (13)	13 (15)	
	Neurological	1 (1.2)	1 (1.2)	0 (0)	0 (0)	0 (0)	0 (0)	
	Heart and vessels	8 (9.3)	2 (2.3)	2 (2.3)	2 (2.3)	2 (2.3)	0 (0)	
	Lung	2 (2.3)	0 (0)	0 (0)	2 (2.3)	0 (0)	0 (0)	
	Cancer	4 (4.8)	0 (0)	1 (1.2)	1 (1.2)	1 (1.2)	1 (1.2)	
	Muskulosceletal	5 (5.8)	0 (0)	3 (3.5)	2 (2.3)	0 (0)	0 (0)	
	Other	9 (10.5)	2 (2.3)	2 (2.3)	2 (2.3)	0	3 (3.5)	
	Multiple	7 (8.1)	0 (0)	5 (5.8)	1 (1.2)	1 (1.2)	0 (0)	

*FAS* Functional Assessment Scale, *IQR* Interquartile range; Group comparisons are completed using Chi-square tests for categorical variables and Kruskall-Wallis for continuous/interval variables, as none of the continuous variables were normally distributed

### Description of healthcare and social support needs, provision and unmet needs

**Fig. 2:**
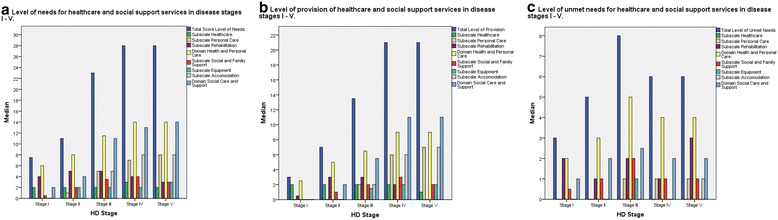
Bargraphs illustrating level of needs, provision and unmet needs for NPCS total, domain and subscale scores

Bargraphs with median values for disease stages I-V for the NPCS total, domain and subscale scores for Needs, Gets and Unmet needs are presented Fig. 2. In general, the median values for NPCS total score and domain scores for Needs and Gets increase from disease stage I – IV, and remain stable from stage IV to V. NPCS Needs and Gets median values for subscales Personal care and Accommodation follow a similar pattern.

Proportions of patients with unmet needs in the total sample and across disease stages are presented in Table 3. Results show high proportions of patients with unmet needs for NPCS total score (92 %), domains Health and personal care (83 %), and Social care and support (79 %), and subscales Rehabilitation (74 %) and Social and family support (66 %). The highest proportion of patients with unmet needs for the overall and domain scores was found in disease stage III (95 % each). Comparing the proportions of patients with unmet needs between stages, significant group differences were found on subscales Personal Care (*p* = 0.00) and Accommodation (*p* = 0.00).

**Table 3:** Proportions of unmet needs among participants

			Complete sample (*N* = 86)	Stage I (*n* = 12)	Stage II (*n* = 23)	Stage III (*n* = 19)	Stage IV (*n* = 15)	Stage V (*n* = 17)	
Variable			n (%)	n (%)	n (%)	n (%)	n (%)	n (%)	*P*-value (2 sided)
NPCS^a^ total score		Met needs	6 (7)	1 (8)	2 (9)	0 (0)	1 (7)	2 (12)	0.723*
		Unmet needs	79 (92)	11 (92)	21 (91)	18 (95)	14 (93)	15 (88)	
Domain score Health and Personal care		Met needs	15 (17)	3 (25)	5 (22)	1 (5)	2 (13)	4 (23.5)	0.502*
		Unmet needs	71 (83)	9 (75)	18 (78)	18 (95)	13 (87)	13 (76.5)	
Subscale	Healthcare	Met needs	59 (69)	10 (83)	18 (78)	11 (58)	9 (60)	11 (65)	0.426*
		Unmet needs	27 (31)	2 (17)	5 (22)	8 (42)	6 (40)	6 (35)	
	Personal Care	Met needs	35 (52)	12 (100)	16 (70)	5 (26)	5 (33)	7 (41)	0.001
		Unmet needs	41 (48)	0 (0)	7 (30)	14 (74)	10 (67)	10 (59)	
	Rehabilitation	Met needs	22 (26)	4 (33)	6 (26)	1 (5)	6 (40)	5 (29)	0.180*
		Unmet needs	64 (74)	8 (67)	17 (74)	18 (95)	9 (60)	12 (71)	
Domain^a^ score Social Care and Support		Met needs	17 (20)	5 (42)	4 (17)	0 (0)	4 (27)	4 (23.5)	0.070*
		Unmet needs	68 (79)	7 (58)	19 (83)	18 (95)	11 (73)	13 (76.5)	
Subscale	Social and family support^a^	Met needs	28 (33)	6 (50)	6 (26)	2 (10.5)	6 (40)	8 (47)	0.098*
		Unmet needs	57 (66)	6 (50)	17 (74)	16 (84)	9 (60)	9 (63)	
	Specialist equipment	Met needs	45 (52)	8 (67)	13 (56.5)	6 (32)	9 (60)	9 (53)	0.310
		Unmet needs	41 (48)	4 (33)	10 (43.5)	13 (68)	6 (40)	8 (47)	
	Accommodation	Met needs	59 (69)	12 (100)	20 (87)	12 (63)	8 (53)	7 (41)	0.002*
		Unmet needs	27 (31)	0 (0)	3 (13)	7 (37)	7 (47)	10 (59)	

*NPCS* Needs and Provision Complexity Scale; Chi-square test for categorical variables was used; *Five or less cells have expected count less than five. ^a^missing data (1 missing NPCS total score, disease stage III; 1 missing domain score social care and support, disease stage III; 1 missing subscale social and family support, disease stage III)

### Factors associated with level of unmet needs across the disease phases (early, middle and advanced)

The results of modelling total level of unmet needs (NPCS total score unmet needs) and level of unmet needs for the two NPCS domains Health and personal care and Social care and support respectively are displayed in Table 4. Being in the middle and advanced phase of HD increased the odds of having a high level of total unmet needs by 3.5 times (OR = 3.5) and 1.4 times (OR =1.4) respectively, whereas the patient reporting their needs without help from an informant tended to decrease the odds of having high level of unmet needs (OR =0.52). Similar results were found for level of unmet needs in the domain Health and personal care: the middle and advanced phase of HD tended to increase the odds of having a high level of unmet needs (OR = 2.77 and OR 2.20 respectively). Additionally, higher education and patients reporting their needs without help from an informant tended to decrease the odds of reporting a high level of unmet needs in this domain (OR = 0.48 and OR = 0.57 respectively). Furthermore, higher education tended to increase the odds of reporting a high level of unmet needs in the domain of Social care and support (OR = 1.3). Having comorbid conditions tended to decrease the odds of reporting a high level of unmet needs for Social care and support (OR = 0.65). Results of residual analyses identified two extreme cases for the model for the Total level of unmet needs. Removing the cases from the analyses did not change the results. No outliers were identified for the level of unmet needs on either of the NPCS domains. Hosmer and Lemeshow tests for goodness-of-fit were satisfactory for all three models.

**Table 4:** Factors associated with level of unmet needs using binary multiple logistic regression models for total level of unmet needs (NPCS total score), level of unmet needs for health and personal care services (NPCS health and personal care score) and for level of unmet needs for social care and support services (NPCS social care and support score)

	NPCS unmet needs: total (*N* = 79)	NPCS unmet needs: domain health and personal care (*N* = 71)	NPCS unmet needs: domain social care and support (*N* = 68)
Socio-demographic and clinical Variables	Multivariate analysis OR (95 % CI)^**^	Multivariate analysis OR (95 % CI)^***^	Multivariate analysis OR (95 % CI)^****^
Age	1.03 (0.98 – 1.08)	1.02 (0.97 – 1.07)	1.00 (0.96 – 1.05)
Education Level	0.78 (0.28 – 2.22)	0.48 (0.16 – 1.45)	1.40 (0.467 – 3.95)
High education vs. lower education^a^			
Disease duration	0.97 (0.86 – 1.10)	0.98 (0.86 – 1.13)	1.00 (0.89 – 1.13)
Comorbidity	0.91 (0.31 – 2.70)	0.83 (0.25 – 2.74)	0.65 (0.22 – 1.90)
Comorbidity vs. no comorbidity^a^			
HD Phase	3.57 (0.89 – 14.4)^*****^	2.77 (0.62 – 12.36)	1.06 (0.27 – 4.18)
Middle phase vs. early phase^a^			
HD Phase	1.38 (0.32 – 6.0)	2.20 (0.42 – 11.67)	1.06 (0.24 – 4.75)
Advanced phase vs. early^a^			
Informant	0.52 (0.13 – 2.04)	0.57 (0.14 – 2.30)	1.03 (0.24 – 3.6)
Patient vs. patient with informant^a^			

^a^Reference group

^*^OR > 1 increase the Odds of having a high level of unmet needs; OR < 1 decrease the odds of having high level of unmet needs

^**^Hosmer and Lemeshow Goodness-of-fit test *χ* ^2^ 9.12; df 8; *P* = 0.332

^***^Hosmer and Lemeshow Goodness-of-fit test *χ* ^2^ 6.63; df 8; *P* = 0.612

^****^ Hosmer and Lemeshow Goodness-of-fit test *χ* ^2^ 6.65; df 8; *P* = 0.575

^*****^Approached significance (*p* = 0.074)

## Discussion

This is the first study to systematically investigate to which extent healthcare and social needs are met in a representative sample of HD patients by using the newly developed Needs and Provision Complexity Scale (NPCS).

As expected, the results indicate a high frequency of unmet needs and gaps in provision of healthcare and social support services at the overall level and in the domains health and personal care and social support services across all five disease stages. More than half of the patients had unmet needs in the NPCS subscales, including Rehabilitation and Social and family support. The results are, in general, in agreement with a previously mentioned survey in the UK [28], and a recent study of HD patients in Europe [29], and thus, may represent the real needs of the HD population.

Contrary to our hypothesis, the results suggest that the most substantial gaps between healthcare and social support service needs and provision are in the middle phase (disease stage III) of HD, in terms of both proportion of patients and level of unmet needs. Patients in this phase represent a heterogenic group, due to higher variation in symptom presentation and progression [31]. A prerequisite for offering adequate help in the middle phase of HD is that healthcare providers understand the needs of these patients and collaborate with family caregivers [32]. Thus, a stronger focus on monitoring patients’ symptoms and functioning is warranted, and healthcare services need to be targeted to this specific group of HD patients. Even though the level of needs for social and healthcare services as a total are greater in disease stages IV and V, these patients receive a higher amount of services, resulting in a smaller amount of unmet needs. This may be due to symptoms being more overt in these stages causing patients to no longer being able to carry out daily activities independently, which may lead to greater awareness of the needs of these patients. Indeed, observations from clinical practice indicate that HD patients in later stages tend to have a higher caregiver frequency and are more often cared for outside the home. Our findings are also in line with research on patient with long-term neurological conditions reporting that patients whose rehabilitation needs were met were more dependent at 12 months after discharge from hospital than those with unmet needs [33]. Furthermore, studies identifying unmet needs after traumatic brain injury indicate that patients with more visible needs have a higher degreee of met needs, which may reflect that health professionals are working actively and responsibly for the patient; thus, the patients are more satisfied and perceive their needs as met [34].

### Factors associated with level of unmet needs across the disease phases

Furthermore, we aimed to assess which socio-demographic and clinical factors were associated with the level of unmet needs for healthcare and social support services. Modelling unmet needs further illustrated the association between disease phase and level of unmet needs for the total level of unmet needs and in the domain Health and personal care. A lower level of education tended to decrease the odds of having a high level of unmet needs in health and personal care domain. One possible explanation for this finding can be related to trends in the general population; people with higher education are more resourceful and have a better understanding and awareness of what healthcare and social support services are available or that they are entitled to, as well as more resources to follow up on receiving the services they need [35]. As a consequence they may report a greater amount of unmet needs. Regardless, we have to interpret this result with caution, as we did not address patients cognitive or behavioral states in this study.

Furthermore, if the survey interview was conducted with the patient only, results showed trends toward decreased odds of having high levels of unmet needs. This may reflect patients’ having reduced awareness of their symptoms causing them to underreport and deny needs for health and personal care services. On the other hand, research has shown that in some cases, proxies tend to overestimate patient disability [36] and hence, may overestimate the patients’ needs for healthcare and social services. This often appears to be the case in clinical practice and the “truth” often seems to be somewhere in the middle.

None of the socio-demographic and clinical factors included were significantly associated with level of unmet needs in the social care and support domain. However, education level tended towards an impact on this domain as higher education increased the chance of having higher levels of unmet needs. A contradiction seemed to emerge with this result as a higher educational attainment, in general, has been associated with higher levels of social support. Future studies are needed to tease this apart, as our data are limited in their ability to provide more insight. Furthermore, comorbidity tended to decrease the chance of having higher levels of unmet needs for social care and support in this study. The presence of comorbid conditions has been associated with poorer social functioning and further research is needed for a fuller understanding of this finding.

Modelling the gaps in healthcare and social support services provide additional support to the overall level of unmet needs and level of unmet needs for health and personal care increasing considerably for patients in the middle phase of HD. As mentioned before, the middle phase of HD represents a largely heterogenic group of patients, and this phase may present the most challenges and unmet needs as the family starts to see an increase of symptoms and the person with HD may have cognitive deficits and struggle to see their own problems. It can be considered a transitional phase, where patients progress from being relatively independent to becoming increasingly dependent in various areas of daily life. The transitional character of this phase may cause additional challenges in providing the tailored multidisciplinary healthcare these patients need. Providing adequate care to patients with HD should include regular monitoring and evaluation of symptoms and functioning from the moment of diagnosis, but may be particularly important in the middle phase. One of the important messages to clinicians is to not only evaluate clinically but also follow up patients and assess needs for healthcare and social services in a systematic manner, in order to provide adequate comprehensive care during the course of disease.

### Limitations and strengths

The present study has some methodological limitations. Firstly, a cross-sectional study design prevents us from discussing any causal relationship between independent variables and NPCS scores representing levels of unmet needs. The results need to be further studied with a longitudinal study design and, ideally, in a larger population of HD patients.

Secondly, the response rate of 54.4 % cases reduced power of our analyses, and some form of population bias cannot be excluded. Patients with reduced self-awareness may not be in contact with healthcare institutions and therefore may have fallen out of reach. If they did receive an invitation they may have declined or chose not to reply because they do not perceive themselves as being ill. Additionally, patients in late stages of the disease are highly dependent on their primary (family) carers who may not have had the time or energy to reply. Yet, considering the clinical picture of HD, the population in this study may be considered representative and our response rate can be considered satisfactory. A strength in the present study is that contrary to many studies (i.e., [29], patients in the advanced phase (stages IV and V) were not underrepresented. However, one of the most important limitations is that behavioral and cognitive domains were not addressed in this study due to the fact that the data were collected by a survey and not by clinical evaluation. Thus, the interpretation of the study results should be made with caution.

The NPCS was not validated for Norwegian language and circumstances. Yet, the instrument has shown good psychometric properties [37] and is originally developed for this type of patient populations. There are currently no other instruments available to assess needs for healthcare and social support services in a similar normative and systematic manner. In order to ensure obtaining best possible reliability of results obtained using the NPCS Scale we discussed each of the NPCS items regarding the interpretation for rating in the context of the Norwegian healthcare system and this particular patient group, in addition to a carefully executed translation process.

### Implications and future directions

The results of the present study suggest that particular focus should be warranted for patients entering the middle phase of HD. Investigating factors associated with the level of unmet needs for healthcare and social support services further suggest that the patients’ education level may be of importance when surveying the needs for healthcare services in HD patients. Also, when discussing needs and provision of services, an informant closely related to the patient should ideally be present. Further studies including a larger population and longitudinal study design should be performed in order to verify the results of the present study and to shed further light on the predictive factors for level of unmet needs. Of particular interest are the needs for social and family support [20]. Moreover, closer investigating the potential influence of cognitive and psychiatric symptoms and self-awareness on levels of unmet needs for healthcare and social support services deserves further research.

## Conclusions

This study indicates unmet needs for health and personal care and social support services among HD patients and across all five disease stages. However, the most substantial gaps in healthcare and social support services were identified in the middle phase (disease stage III) of HD (in terms of both proportion of patients and level of unmet needs). One important message to clinicians is to not only evaluate the patients clinically, but also follow up patients and assess needs for healthcare and social services in a systematic manner, in order to provide adequate comprehensive care during the course of disease.

## Additional file

Additional file 1:
**The Needs and Provision Complexity Scale.** (PDF 102 kb)
