# Supplementary material for: Unmet needs for healthcare and social support services in patients with Huntington’s disease: a cross-sectional population-based study
Source: Orphanet J Rare Dis. 2015 Sep 28;10:124. doi: 10.1186/s13023-015-0324-8 (PMC4585992; doi:10.1186/s13023-015-0324-8)
Supplement: Additional file 1: — The Needs and Provision Complexity Scale. (PDF 102 kb) [file 13023_2015_324_MOESM1_ESM.pdf]

King's College London
University of London

The North West London Hospitals NHS
NHS Trust

The Needs & Provision Complexity Scale (NPCS) for LTNC

Extended version

The NPCS can be used and copied freely,

but please acknowledge the originators in all publications

Further information and advice may be obtained from:

Professor Lynne Turner-Stokes DM FRCP
Herbert Dunhill Chair of Rehabilitation, King's College London.

Regional Rehabilitation Unit,
Northwick Park Hospital,
Watford Road,
Harrow, Middlesex.
HA1 3UJ

Tel: +44 (0) 208-869-2800;
Fax: +44 (0) 208-869-2803
Email: lynne.turner-stokes@dial.pipex.com

Background

The National Service Framework (NSF) for Long Term Neurological Conditions (LTNC) promotes joined-up services to provide holistic, person-centred care (Department of Health, 2005). It includes 11 quality Requirements covering the care pathway from diagnosis to death. Critically, integrated care planning (QR1) provides the backbone to the NSF recommendations. Figure 1 illustrates how these elements fit together to provide life long support for people with LTNC. It is recognised, however, that resources to support integrated care planning are currently very limited, and this presents a major threat to implementation of the NSF recommendations. It is vital therefore to be able to evaluate service provision in relation to need, both at an individual and at a population level, in order to focus future service development efficiently.

LTNC are themselves a highly diverse group of conditions, and within those, people have widely different needs for services, against which the adequacy of service provision must be judged. Diagnosis is a poor determinator of need in this context, and we require some other way of defining need for services. The Needs and Provision Complexity Scale (NPCS) to measure needs for community care and rehabilitation and to assess provision against these needs.

The Needs and Provision Complexity Scale (NPCS)

The NPCS is an ordinal scale with five main domains and fifteen subscales
It has a total range of 0-50

Domains	Range	Items		Range
Healthcare	0-6	Medical care needs		M 0-3
		Skilled nursing needs		N 0-3
Personal care	0-10	Number of carers		CN 0-2
		Care frequency		CF 0-5
		Personal assistant / enabler		PA 0-3
Rehabilitation	0-9	Therapy disciplines		TD 0-3
		Therapy Intensity		TI 0-3
		Vocational support/rehabilitation		VR 0-3
Social and family	0-13	Social work case management		S 0-3
Support		Family carer support		FC 0-3
		Respite - residential		RR 0-3
		Respite - day care		RD 0-2
		Advocacy		AD 0-2
Environment	0-12	Equipment		E 0-3
		Accommodation		AC 0-9
Total	0-50			

The NPCS is divided into to parts

Part A: Needs – defines the needs for input under the different headings
(The NPCS records both professional and personal perspectives on what the patient needs (professional view) and what they would like (personal choice).
Part B: Provision – defines the current level of input or service currently provided within the same structure.
Unmet needs – are therefore recorded as the difference between the two scales.

Broadly, the rating levels under each item are designed to determine a) the number of staff required and b) the frequency of needs/intervention. Level descriptors give a rough guide as to what might "occasional", "regular" or "frequent" , but these are not designed to be strictly defined cut-off points.

The summary score sheet includes a check list of the specific services required under each heading It also includes a set of boxes to record the reasons for variance (unmet need) which may be
a) service not available – ie there is no service available, or it has not been offered
b) service declined – ie service has been offered, but declined by the pt / carer (this will often be because they consider that which has been offered to be unsuitable) – there is space to record the specific circumstances if desired.
c) other - some other reason

The Needs & Provision Complexity Scale (NPCS) for LTNC

PATIENT IDENTIFICATION		
**Name:**	**NHS No:**	**Date of score:...../...../.......**

PART A - NEEDS: For each subscale, circle highest level applicable

1. MEDICAL CARE NEEDS – requiring intervention from a doctor for **investigation, monitoring or treatment**

- Specialist Medical input may be from any medical specialty

**M 0**	**GP occasional –**- no regular contact – self-initiated visits to GP as required	Types of medical care<br>☐ Neurology<br>☐ Rehab medicine<br>☐ Neuropsychiatry<br>☐ Palliative care<br>☐ Other
**M 1**	**GP active monitoring -** regular monitoring/ treatment by GP solely	
**M 2**	**Low level specialist support** eg for largely stable condition<br>On-going monitoring/ treatment by GP with occasional specialist advice / review	
**M 3**	**Active specialist medical intervention required** eg for changing/unstable condition or for unresolved symptoms.<br>Investigation or treatment requiring frequent contact with specialist medical team	

2: SKILLED NURSING NEEDS – intervention required from trained and/or specialist nursing staff

eg district nursing or Specialist nurse (E.g. for wound care, bladder / bowel management / medication monitoring / specialist advice/support/counselling)

**N 0**	**No needs** for skilled nursing	Types of nursing care<br>☐ District nurse<br>☐ Specialist nurse<br>☐ Neurology<br>☐ Mental Health<br>☐ Palliative care<br>☐ Other....
Requires **intervention** from a **trained or specialist nurse:**		
**N 1**	**Occasional intervention** (eg **monthly or less)**	
**N 2**	**Regular intervention** eg every **1-2 weeks**	
**N 3**	F**requent intervention** on a **daily** basis, or **Several times a week**	

3. PERSONAL CARE - In and around the home.

3a: Number of Carers: Required to help with basic self-care

**CN 0**	**No carers** required for basic care activities	Who provides this help?:<br>☐ Informal family care<br>☐ Formal paid carers<br>☐ Other......................................
**CN 1**	Requires **help from 1 person** for most basic care needs	
**CN 2**	Requires **help from ≥2 people** for most basic care needs	

3b: Care frequency: Frequency of care for help with basic self-care, including maintaining safety

**CF 0**	**No need** for help with self care.
**CF 1**	**Occasional need** – less than daily for help with self care, or extended activities of daily living
**CF 2**	Requires **regular help once daily**
**CF 3**	Requires **regular help 2-3 times** a day – could be met by an intermittent visiting care package<br>Able to be left safely for >4 hours and does not require care / supervision at night
**CF 4**	**Frequent or unpredictable care needs**, requiring the presence of someone most of the time<br>Cannot be left safely for >4 hours or requires care / supervision at night (but not waking night care)
**CF 5**	**Requires constant supervision -** unable to be left alone in the house, even for short periods<br>AND/Or requires **waking night care –** needs > 2 interventions at night)

**3c: Personal assistant/enabler** Frequency of assistance for participation in day time community activities	
**PA 0**	**No need** for assistance with community activities
**PA 1**	**Occasional need** – 1-2 days per week
**PA 2**	**Frequent need** – 3-5 days/week
**PA 3**	**Daily** – 6-7 days/week

4. THERAPY NEEDS – including outpatient, community-based and vocational rehabilitation

**Number of Therapy Disciplines: -** required to be actively involved in treatment (ie at least 1 hr per month)		
**TD 0**	**0**	**Tick therapy disciplines required:**
**TD 1**	**Single discipline only**	☐ Physio ☐ O/T ☐ SLT ☐ Dietetics ☐ Orthotics / Prosthetics
**TD 2**	**Individual disciplines,** not co-ordinated	☐ Psychology ☐ Counselling ☐ Mental health ☐ Other:
**TD 3**	**Co-ordinated interdisciplinary team**	

**Therapy Intensity: -** Overall intensity of trained therapy intervention required	
**TI 0**	**No need** for trained therapy intervention
**TI 1**	**Requires occasional review or maintenance** programme – **OR** requires **Group therapy solely**<br>Eg Rehab needs met by family/care staff or self-exercise, supervised by therapist eg 1-2 hrs total/month
**TI 2**	**Regular intervention** for maintenance / treatment eg **every 1-2 weeks**: OP or domiciliary treatment
**TI 3**	**Requires frequent intervention** involving several sessions per week

5. VOCATIONAL /EDUCATIONAL SUPPORT NEEDS

**VR 0**	**No need** for vocational/educational support
**VR 1**	**Requires vocational assessment / advice** or **educational statementing**
**VR 2**	**Requires on-going vocational /educational support** eg Access to work scheme, or withdrawal from work
**VR 3**	**Requires formal vocational / educational rehab** eg work prep, work re-training, supported placements

6. SOCIAL WORK AND CASE MANAGEMENT -– support / intervention to co-ordinate care / services

**S 0**	**No needs** for social work or case management
**S 1**	Requires **occasional intervention or available for advice** when needed eg contact **2-3 x per year**
**S 2**	Requires **regular intervention or contact** eg every **1-2 months**
**S 3**	Requires **frequent intervention or contact** eg every **1-2 weeks**

7. FAMILY / CARER SUPPORT / RESPITE NEEDS

**7a: Family career support**	
**FC 0**	**No needs** for family / carer support
**FC 1**	**Assessment required** for family / carer
**FC 2**	**Time-limited family/carer support required** eg for skills training
**FC 3**	**On-going family/carer support required** eg for emotional support

7b. Respite - residential and day care centre:

**RESIDENTIAL RESPITE**		
**RR 0**	**No need** for residential respite care	**Type of respite care**<br>☐ Home-based temporary live-in care<br>☐ Residential home<br>☐ Nursing home<br>☐ Specialist nursing home<br>☐ Hospice<br>☐ Other
**RR 1**	Requires **occasional residential respite –** eg to cover holidays etc.	
**RR 2**	Requires **regular planned residential respite ,** but not very frequent (eg 1-2 weeks per 6 months))	
**RR 3**	Requires **frequent planned residential care** (eg every 4-6 weeks) AND/OR **back-up support at times of crisis**	
**DAY CARE**		
**RD 0**	**No need** for day care	**Type of day care**<br>☐ Community day centre<br>☐ Specialist day centre<br>☐ Hospice
**RD 1**	**Occasional day care** – 1-2 days per week	
**RD 2**	**Frequent day care** – 3-5 days/week	

8. ADVOCACY NEEDS --

**AD 0**	**No needs** for advocacy
**AD 1**	**Mental capacity assessment** required
**AD 2**	**Independent advocacy** required

9. SPECIALIST EQUIPMENT – Eg Special seating, assistive technology, ventilation equipment

**E 0**	**No specialist equipment required**	**Types of Equipment**<br>☐ Basic lifting handling equipment<br>☐ Seating/wheelchair<br>☐ Standing/postural support<br>☐ Electronic Assistive technology<br>☐ Communication aid<br>☐ Assisted ventilation<br>☐ Other..
**E 1**	**Basic equipment required (**eg from social services equipment store eg kitchen aids, commode, bed, hoist etc)	
**E 2**	**Specialist equipment required –** equipment requiring professional assessment and provision (eg seating, standing frames)	
**E 3**	**Highly specialist equipment required –** bespoke equipment requiring professional prescription (eg environmental control, communication aids, ventilatory support)	

10. ACCOMMODATION NEEDS

**AC 0**	**No need for special accommodation**
**AC 1**	**Restricted accommodation options** (eg requires ground floor or lift access accommodation)
**AC 2**	**Requires partially adapted accommodation** (eg rails, ramps etc)
**AC 3**	**Requires fully adapted accommodation** (eg fully wheelchair accessible)
**SHELTERED AND RESIDENTIAL CARE**	
**AC 4**	**Requires sheltered living accommodation** (eg warden controlled)
**AC 5**	**Requires supervised living arrangement eg small group home**
**AC 6**	**Requires residential care home setting**
**AC 7**	**Requires nursing home care**
**AC 8**	**Requires specialist nursing home**
**AC9**	**Requires Hospice care**

Part B: The Inputs provided

Part B is intended to mirror Part A,

except that it records what the person actually gets –

and so identifies <u>unmet need</u>

PART B – The Inputs Provided

PATIENT IDENTIFICATION		
**Name:**	**NHS No:**	**Date of score:...../...../.......**

For each subscale, circle <u>highest level</u> applicable

1. MEDICAL CARE PROVISION – intervention from a doctor for **investigation, monitoring or treatment**

- Specialist Medical input may be from <u>any</u> medical specialty

		Types of medical care
**M 0**	**GP occasional** –- no regular contact – self-initiated visits to GP as required	☐ Neurology<br>☐ Rehab medicine<br>☐ Neuropsychiatry<br>☐ Palliative care<br>☐ Other
**M 1**	**GP active monitoring -** regular monitoring/ treatment by GP solely	
**M 2**	**Low level specialist support** eg for largely stable condition<br>On-going monitoring/ treatment by GP with occasional specialist advice / review	
**M 3**	**Active specialist medical intervention** eg for changing/unstable condition or for unresolved symptoms.<br>Investigation or treatment requiring frequent contact with specialist medical team	

2: SKILLED NURSING PROVISION – intervention from trained and/or specialist nursing staff

eg district nursing or Specialist nurse (E.g. for wound care, bladder / bowel management / medication monitoring / specialist advice/support/counselling)

		Types of nursing care
**N 0**	**No provision of** skilled nursing	☐ District nurse<br>☐ Specialist nurse<br>☐ Neurology<br>☐ Mental Health<br>☐ Palliative care<br>☐ Other....
**N 1**	**Occasional intervention** from a **trained or specialist nurse** (eg **monthly or less)**	
**N 2**	**Regular intervention** from a **trained nurse or specialist nurse** eg every **1-2 weeks**	
**N 3**	**Frequent intervention** from a **trained nurse or specialist nurse** on a **daily** basis, or **Several times a week**	

3. PERSONAL CARE - In and around the home.

3a: Number of Carers: provided to help with <u>basic self-care</u>

		Who provides this help?:
**CN 0**	**No carers** for basic care activities	☐ Informal family care<br>☐ Formal paid carers<br>☐ Other......................................
**CN 1**	**1 carer**	
**CN 2**	**2 carers**	

3b: Care frequency: Frequency of care for help with <u>basic self-care</u>, including maintaining safety

**CF 0**	**No provision** for help with self care.
**CF 1**	**Occasional care visits** – less than daily for help with self care, or extended activities of daily living
**CF 2**	**Once daily care visit** 1-2 hours
**CF 3**	**2-3 care visits per day** – (or 3-6 hours care per day in total) - no night time care
**CF 4**	**Live-in or all day care package** - >6 hours
**CF 5**	**1:1 care throughout the day** <u>AND/Or</u> **waking night care**

**3c: Personal assistant/enabler - Assistance for participation in day time community activities**	
**PA 0**	**No provision** for assistance with community activities
**PA 1**	**Occasional assistance provided** – 1-2 days per week
**PA 2**	**Frequent assistance provided** – 3-5 days/week
**PA 3**	**Daily assistance provided** – 6-7 days/week

4. THERAPY PROVISION – including outpatient, community-based and vocational rehabilitation

**Number of Therapy Disciplines: -** actively involved in treatment (ie at least 1 hr per month)			
**TD 0**	**0**	**Tick therapy disciplines involved:**	
	**Single discipline only**	☐ Physio<br>☐ O/T	☐ Psychology<br>☐ Counselling
**TD 2**	**Individual disciplines,** not co-ordinated	☐ SLT<br>☐ Dietetics	☐ Mental health<br>☐ Other:
**TD 3**	**Co-ordinated interdisciplinary team**	☐ Orthotics / Prosthetics	

**Therapy Intensity: -** Overall intensity of trained therapy intervention	
**TI 0**	**No therapy intervention** (or <1 hr per month)
**TI 1**	**Occasional review or maintenance** programme –- about **1-2 hours/month** in total – **OR** attends for **Group therapy solely**
**TI 2**	**Regular intervention** for maintenance / treatment eg **every 1-2 weeks**:
**TI 3**	**Frequent intervention** involving **several sessions per week** (may be from different disciplines)

5. VOCATIONAL /EDUCATIONAL SUPPORT PROVISION

**VR 0**	**No provision** for vocational/educational support
**VR 1**	**Received/ing vocational /educational assessment / advice** or **statementing**
**VR 2**	**Receives on-going vocational/educational support** eg Access to work scheme, or withdraw from work
**VR 3**	**Receives formal vocational / educational rehabilitation** eg work preparation, work re-training, supported placements

6. SOCIAL WORK AND CASE MANAGEMENT -- support / intervention to co-ordinate care / services

**S 0**	**No provision** of social work or case management – **or very inconsistent** (ie effectively none)
**S 1**	**Occasional intervention or contacts for advice** when needed eg **2-3 times per year**
**S 2**	Re**gular intervention or contact** eg every **1-2 months**
**S 3**	F**requent intervention or contact** eg every **1-2 weeks**

7. FAMILY / CARER SUPPORT / RESPITE PROVISION

**7a: Family career support**	
**FC 0**	**No provision** for family / carer support
**FC 1**	**Received/ing assessment** for family / carer
**FC 2**	**Received/ing family/carer support** eg for skills training
**FC 3**	**Receives on-going family/carer support** eg for emotional support

7b. Respite - residential and day care centre:

**RESIDENTIAL RESPITE**		
**RR 0**	**No provision** for residential respite care	**Type of respite care**<br>☐ Home-based temporary live-in care<br>☐ Residential home<br>☐ Nursing home<br>☐ Specialist nursing home<br>☐ Hospice<br>☐ Other
**RR 1**	**Occasional residential respite provision–** eg to cover holidays etc.	
**RR 2**	**Regular planned residential respite provision ,** but not very frequent (eg 1-2 weeks per 6 months))	
**RR 3**	**Frequent planned residential care** (eg every 4-6 weeks) AND/OR **back-up support at times of crisis**	
**DAY CARE**		
**RD 0**	**No provision** for day care	**Type of day care**<br>☐ Community day centre<br>☐ Specialist day centre<br>☐ Hospice
**RD 1**	**Occasional day care provided** – 1-2 days per week	
**RD 2**	**Frequent day care provided** – 3-5 days/week	

8. ADVOCACY PROVISION --

**AD 0**	**No provision** for advocacy
**AD 1**	**Received/ing mental capacity assessment**
**AD 2**	**Receiving Independent advocacy**

9. SPECIALIST EQUIPMENT – **Eg Special seating, assistive technology, ventilation equipment**

**E 0**	**No specialist equipment /provision inadequate**	**Types of Equipment**<br>☐ Basic lifting handling equipment<br>☐ Seating/wheelchair<br>☐ Standing/postural support<br>☐ Electronic Assistive technology<br>☐ Communication aid<br>☐ Assisted ventilation<br>☐ Other..
**E 1**	**Basic equipment provided (**eg from social services equipment store eg kitchen aids, commode, bed, hoist etc)	
**E 2**	**Specialist equipment provided –** equipment requiring professional assessment and provision (eg seating, standing frames)	
**E 3**	**Highly specialist equipment provided –** bespoke equipment requiring professional prescription (eg environmental control, communication aids, ventilatory support)	

10. ACCOMMODATION PROVISION

**AC 0**	**No provision for special accommodation**
**AC 1**	**Restricted accommodation options met** (eg requires ground floor or lift access accommodation)
**AC 2**	**Has partially adapted accommodation** (eg rails, ramps etc)
**AC 3**	**Has fully adapted accommodation** (eg fully wheelchair accessible)
**SHELTERED AND RESIDENTIAL CARE**	
**AC 4**	**Has sheltered living accommodation** (eg warden controlled)
**AC 5**	**Has supervised living arrangement eg small group home**
**AC 6**	**Has residential care home setting**
**AC 7**	**Has nursing home care**
**AC 8**	**Has specialist nursing home**
**AC9**	**Has Hospice care**
